# Asbestos Air Pollution: Description of a Mesothelioma Cluster Due to Residential Exposure from an Asbestos Cement Factory

**DOI:** 10.3390/ijerph17082636

**Published:** 2020-04-12

**Authors:** Luigi Vimercati, Domenica Cavone, Maria Celeste Delfino, Antonio Caputi, Luigi De Maria, Stefania Sponselli, Vincenzo Corrado, Giovanni Maria Ferri, Gabriella Serio

**Affiliations:** 1 Interdisciplinary Department of Medicine, Occupational Medicine “B. Ramazzini”, University of Bari Medical School, 11 G, Cesare Square, 70124 Bari, Italy; domenica.cavone@uniba.it (D.C.); maria.delfino@uniba.it (M.C.D.); antonio.caputi@uniba.it (A.C.); luigi.demaria@uniba.it (L.D.M.); stefania.sponselli@uniba.it (S.S.); vincenzo.corrado@uniba.it (V.C.); giovannimaria.ferri@uniba.it (G.M.F.); 2 Department of Emergency and Organ Transplantation (DETO), Pathology Division, University of Bari Medical School, 11 G, Cesare Square, 70124 Bari, Italy; gabriella.serio1@uniba.it

**Keywords:** asbestos, asbestos cement factory, mesothelioma, air pollution, residential–environmental exposure, southern Italy, mesothelioma register

## Abstract

The study describes a cluster of 71 malignant mesothelioma cases among Bari residents without asbestos exposure other than residential exposure. This small cohort, as expected, was composed of a majority of females (56.34%) with a M/F ratio of 0.8, ages ≤ 65 years old (52.11%) and the epithelioid morphological type (78.87%). Sixty-four subjects (90.14%) lived between 10 m and 1000 m from the asbestos cement factory (Fibronit), and the latency length was longer than 55 years for 25 subjects (35.21%). The adjusted risk (adjusted OR) of observing the epithelial form of mesothelioma among subjects living at small distances from Fibronit was high (OR = 1.870 (0.353–9.905)) for people living 550–1000 m from the site and for those living less than 550 m from the site (OR = 1.470 (0.262–8.248)). Additionally, the subjects with a high length of exposure showed a relevant risk of epithelioid mesothelioma both for 21–40 years of exposure (OR = 2.027 (0.521–7.890)) and more than 40 years of exposure (OR = 2.879 (0.651–12.736)). All of the estimates were high but not significant because this *transitional* study has a typically low power. The adjustment for latency showed the same trend. Using detailed information collected by the regional mesothelioma registry, this study provided evidence of a continuing health impact of the Fibronit asbestos cement factory in Bari on the resident population.

## 1. Introduction

“Asbestos is the generic commercial designation for a group of naturally occurring mineral silicate fibres of the serpentine and amphibole series. These include the serpentine mineral chrysotile (also known as “white asbestos”) and the five amphibole minerals—actinolite, amosite (also known as “brown asbestos”), anthophyllite, crocidolite (also known as ”bleu asbestos”) and tremolite.” [1].

Asbestos, as a pollutant in the air, may be a serious health problem globally, similar to many other environmental pollutants [2,3,4,5,6].

In Italy, even more than 25 years after the ban [7], the general population may be exposed to asbestos even for decades in areas where the reclamations were not completed after the ban. Thus, subjects continue to be exposed, such as construction workers, who must operate in old and unclaimed structures [8]. The relationship between the presence of asbestos fibers in breathed air and malignant mesothelioma (MM) has been shown [1].

Environmental exposure (EE) is defined as neighborhood or residential exposure resulting from outside air pollution and is associated with living in proximity to industrial sources of fiber emissions, naturally occurring asbestos (NOA) or erosion of materials containing asbestos (MCA).

“Environmental asbestos exposure is the exposure to asbestos of the general population in daily living settings…. It can be categorized as follows: exposure seen in residents living a few kilometers away from asbestos mines or factories (neighborhood exposure)….” [7]. That is, EE concerns the exposure deriving from urban air pollution.

Numerous scientific papers have analyzed the association between EE and the onset of mesothelioma [7,9,10,11,12,13,14,15,16,17,18,19,20,21,22,23,24,25].

The most recent reviews and meta-analyses on EE and MM reported meta-relative risks (meta-RRs) of 6.9 (95% CI 4.2–11.4),with trends in risk in relation to fibre type for chrysotile, mixed and amphibole fibres, respectively, meta-RRs were 3.8 (95% CI 0.4 to 38.4), 8.4 (95% CI 4.7 to 14.9) and 21.1 (95% CI 5.3 to 84.5) [17] and a summary relative risk estimate (SRRE) of 5.33 (95% CI 2.53–11.23) from twenty case control and seven cohort studies, including some of those mentioned above [20].

Nonoccupational asbestos exposure (household, domestic and neighborhood) has been identified as an important risk factor and remains the main cause of MM in women [17,20,26,27,28,29].

In Italy, due to long-term asbestos use and the excess of MM incidence and mortality, as well as asbestos exposure in occupational settings and diffuse environmental exposure in the neighborhoods of industrial settings [30], national priority contaminated sites (NPCSs) were identified for land reclamation [31,32]. Moreover, in Italy, an epidemiological surveillance system (ReNaM) has been implemented by means of a national register with regional operating centers. In our region, Apulia southern Italy, the Regional Operational Centre of the National Mesothelioma Register was formed in 1988.

In Bari, the capital town of Apulia, an asbestos cement factory (Fibronit) was documented to have exhibited 50 years of amphiboles (amosite and crocidolite 20%) and chrysotile (80%) use from 1934 to 1985; however, this factory was previously located outside the urban area during the early 1930s and 1940s. Later, the urban expansion in the 1950s reduced the distance from the residential areas, and the factory became completely within the urban perimeter, and houses were built in front of the factory at a distance of the only roadway. Therefore, the approximately 100,000 square meter factory was inside the urban area and bordered three densely populated neighborhoods with 59,716 inhabitants according to the 2011 census survey [33,34]. Site remediation was completed only in 2018.

The aim of this study was to update previous studies and describe the cluster of MM cases among Bari residents with no asbestos exposure other than residential exposure.

## 2. Materials and Methods

The Apulia Mesothelioma Registry (AMR) was established in 1988 and is a population-based registry that is part of a national network (Registro Nazionale Mesoteliomi, (ReNaM) as a regional operating center). ReNaM is a national epidemiological surveillance system for mesothelioma incidence that has been active with force of law since 2002 and is used for the collection of cases and information on previous exposure to asbestos.

The AMR collects all MM cases diagnosed in people living in the region (4,029,053 inhabitants on 1 January 2020) at the time of diagnosis. AMR enlists cases not only with reports mandatory by law as passive research but also with active research from hospital departments involved in the diagnosis (occupational medicine, thoracic surgery, pneumology, pathological anatomies, etc.). All cases in the register had a histological diagnosis confirmed by immunohistochemistry according to the WHO 2015 guidelines [35]. The differential diagnosis, as suggested, must be carried out by the combined use of a minimum of two mesothelial markers and two carcinoma markers. The cases analyzed in this study were all reviewed according to the aforementioned WHO guidelines for maximum diagnostic assessment of the diagnosis.

The exposure is reconstructed and classified using a nationally standardized questionnaire according to national guidelines available online [36]. The occupational, residential and life history of each case is investigated. The questionnaire provides for the complete reconstruction of the familiar history of the subject, including the occupational histories of their spouse, siblings and parents, and their personal history from birth to any military service, residential locations, and working and outside-work history up to the time of mesothelioma diagnosis. Information related to previous exposure to asbestos is collected by a skilled interviewer either directly by the patient if he is still alive or by his closest relatives if he has died. The maximum diagnostic assessment of the exposure is carried out by an industrial hygienist who analyzes the data using a national reference standard developed for the evaluation, with homogeneous criteria including the presence or absence of exposure to asbestos. This phase is preceded by the evaluation and critical review of the questionnaire to verify the completeness and reliability of the information acquired. The exposure classification is qualitative and may result as occupational (definite, probable, possible) or non-occupational. The latter classification includes environmental exposure (due to the residence being near a source of asbestos pollution without work-related exposure), familial exposure (when patients have lived with a cohabitant occupationally exposed and, e.g., from contaminated clothes), domestic exposure (e.g., ironing on asbestos boards, repair activities in the house, use of talc for intimate hygiene, use of asbestos containing material within the house), and leisure activity exposures (other nonoccupational exposures such as those due to leisure-time activities). Information on residential location includes the address throughout the life of the individual, together with the date of taking up and leaving residence at each address and a description of each dwelling and its neighborhood environment.

Informed and written consent was obtained from all participants. All subjects were informed that data from the research protocol would be treated in an anonymous and collective way, with scientific methods and for scientific purposes in accordance with the principles of the Helsinki Declaration.

### 2.1. The MM Cases

The records of MM cases (2236, of which 1620 were males (72.4%) and 616 were females (27.5%)) listed in the AMR from 1989 to 2019 were analyzed. The information in the database allows the tracking of clusters of MM subjects who had ever lived in Bari (436, of which 300 were males (68.8%) and 136 were females (31.2)). For 91% of the cases registered among the residents (396), it was possible to reconstruct the exposure. Fifty-eight percent of the reconstructed cases had occupational exposure, and 2.5% of the family/domestic or outside-work exposure occurred as a result of activities carried out in people’s spare time. Eight percent were classified as unlikely exposure; in seven percent of the registered cases, it was not possible to reconstruct the exposure to asbestos. Lastly, 24.5% of the residents had environmental exposure. A total of 125 cases with environmental exposure opportunities due to the presence of the Fibronit factory or the Rossani military barracks, another source of asbestos pollution in the town, were recorded among the Bari residents. Of these 125 cases, 57% were classified as environmental exposure in the absence of other exposures. Conversely, 43% of the subjects who also had occupational exposure, despite having residences near the two sources of exposure, were not classified as environmental exposures since they were multiple exposures. The seventy-one cases attributable to the Fibronit plant were all subjects without any occupational, nonoccupational familial, domestic or leisure exposure.

### 2.2. Classification of Environmental Asbestos Exposure Associated with the Asbestos Cement Factory and Georeferencing

The attribution of the residence address to each individual was made according to two criteria: considering the prevalent residence (i.e., the address where the individual lived for the longest time during her/his life) and the residence closest to the source of environmental risk (i.e., the address closest to the AC factory). Both references are useful because the duration, as well as the intensity of exposure, influence the probability of contracting MM.

In any case, the residence addresses over the last ten years before the date of diagnosis were excluded, in view of the minimal latency period of the disease between first exposure to asbestos and diagnosis. All addresses were georeferenced as geographic coordinates using the Spotfire Location Analytics GIS tool for graphic representation and calculation of the distances [https://www.tibco.com/products/tibco-spotfire/location-analytics].

There were no historical environmental data on asbestos contamination in the city of Bari.

Environmental asbestos exposure was examined in relation to the history of the remediation of the Fibronit site.

After the plant closed in 1985, the Bari Public Prosecutor’s Office decreed the seizure of the Fibronit area in 1995 for evidentiary purposes and provided technical advice from the office.

In 2001, the site was included among the national priority contaminated sites (NPCSs). Moreover, in 2001, provisional safety began with the removal of the shed covers, removal from the ground and painting with fixative to prevent asbestos fibers from continuing to disperse in the air.

In 2002, the Bari Public Prosecutor’s Office seized the polluted area, still recognizing the presence of “Serious Risks for Public Health”.

In 2005, a construction site for emergency safety works was opened, which was completed in 2007. 

In 2016, the definitive remediation of the Fibronit site began.

In 2018, the remediation work was completed, and the procedure for the construction of a public garden that was to be built in place of the factory simultaneously began.

Regarding the environmental measurements of airborne fibers, the consultant in charge of the 2009 judgment by the court of Bari at the time made the following statement [37]: “the concentration of asbestos fibers was … with a maximum tip of 38.8 fiber–cc during the operation of the mill with the door open. …, the atmospheric concentration of the dust was 2 to 8 times higher than the limit of 176 pp.cc indicated for asbestos by the ACGIH until 1968.”

The statement from 2009 included the following:

“The performance of the abatement systems existing at the time could not be considered sufficient given that the outlet fumes, especially in the compound, were even visible to the naked eye;……. since the plant was surrounded by numerous residential buildings of even 4 and 5 floors,…. For the exchange of air, more than 100,000 m^3^/h of air exited and entered the area; most of the fibers were thus dragged outside with the exchange of air with the consequent pollution of the atmosphere.”

Between 1970 and 1974, some industrial hygiene investigations were carried out with measurements of the concentrations of airborne fibers ranging between 5 ff/l and 20 ff/l, with the highest values measured in the milling department and the pipe and monolithic glass department. Fiber concentrations and worker exposure in previous decades were, in all likelihood, higher than those measured during this period. The operations that resulted in increased exposure to asbestos were emptying and flapping of the bags, grinding, turning of the pipes and cutting of the plates.

In the 1970s, the ACGIH occupational exposure limit (TLV) was 5 ff/cc (fibers/cubic centimeters). The first survey carried out by the ENPI in 1970 found concentrations of up to 20 ff/cc near the mill.

In the second survey carried out in 1972 by the Labor Inspectorate, the maximum concentrations reached were 10 ff/cc near the mill and in the TBM department. Finally, in 1974, an office report prepared by the judiciary carried out withdrawals on three consecutive days, finding concentrations that ranged between 4 and 19 ff/cc in the areas where the riskiest operations were carried out [38].

A statement from 16 September 2009 also stated the following:

“Overall, there is an area (approximately 10 hectares) characterized by extremely high environmental risk. In particular, very large areas have been observed in which the coverage of the countryside level is made up of a centimeter layer of asbestos fibers combined with scarce quantities of cement. This mantle exposed to atmospheric agents has a high degree of alteration with a high dispersion capacity of the mineral. The state of environmental degradation and asbestos pollution of the surrounding sites of the plant was alarming. In the Regional Register of Mesotheliomas RE.Na. M/C.O.R. Apulia, other cases of mesothelioma have arisen among individuals residing near the Fibronit plant. The data related to the average latency period of the disease therefore confirm the hypothesis that the total reconstructed cumulative dose, to which the resident subjects may have been exposed within 500/1000 m from the establishment, may have been higher than the reconstructed cumulative dose to which Fibronit workers who subsequently developed mesothelioma were exposed.”

It should also be reported that in 1974, during the ex officio appraisal ordered by the judiciary in the context of the dispute promoted by workers of Fibronit, the occupational disease asbestosis was recognized. One of the experts examined the wife of a worker also suffering from asbestosis. The woman reported both an exposure to the dusty work clothes that her husband had brought home and to the white snow that she removed daily from the mosquito nets around their home installed for defense against it. The subjects lived in one of the buildings built around the factory [33]. In the same years, environmental sampling carried out in the urban area adjacent to the factory was carried out in places far from sliding roads and therefore was only minimally influenced by car traffic. The results reported values of average concentrations equal to 16.06 × 10^−4^ particles smaller than 5 microns per cubic centimeter (cc) of air [39]. In the paper, the author noted how exposure in urban areas, unlike what happens for occupational exposures, must be considered 24 h a day.

Finally, in 2011, the Bari Court of Appeal ascertained the causal link between the presence of sheds and pleural mesothelioma when confirming the penalty for manslaughter of twelve workers imposed on a former manager, which occurred in a woman who lived a few hundred meters from the factory for over 39 years [40].

In light of these data, it can be estimated that in the volume of air breathed daily (15 breaths per minute x the current volume 0.7 L of air equal to 10.5 L inhaled in one minute, or 10.5 L × 1440 min per day) of 15,120 L, the inhabitants around the factory could have inhaled 24,282.72 fibers [2].

We considered wind and weather patterns to determine the expected dispersion of asbestos fibers from the plant. A preliminary analysis of the prevalent wind direction and force from 1961 to 2010 was performed according to data provided by the Station Bari-Palese Air Force National Centre for Meteorology and Climatology Aeronautics. Monthly frequency distributions of winds at ground level for each synoptic hour (00, 03, 06, 09, 12, 15, 18, 21) were considered. No evidence of the prevalent wind direction was found, as emerged in a previous study [34].

Environmental asbestos exposure was examined, on a case-by-case basis in relation to the linear distance of each residence from the factory, taking into account the duration of exposure and the age at first exposure.

### 2.3. Statistical Methods

We performed univariate, multivariate and cluster analysis.

Stata 15 software was used for analysis (StataCorp. Stata: Release 15. Statistical Software. College Station, TX, USA: StataCorp LP; 2017).

## 3. Results

### 3.1. Cohort Description

The most important characteristics of this small cohort of subjects affected by MM with residential-environmental exposure are related to sex, with a majority of females (56.34%), ages ≤ 65 years old (52.11%), the morphological type with the epithelioid form representing the majority (78.87%), the distance from Fibronit, with 33 subjects (46.47%) living at a distance between 550 m and 1000 m from the plant and only 9.85% of subjects living at a distance of more than 1000 m, exposure length and latency length, which were equally distributed (Table 1) (Supplementary Materials Tables S1–S3).

**Figure 1:**
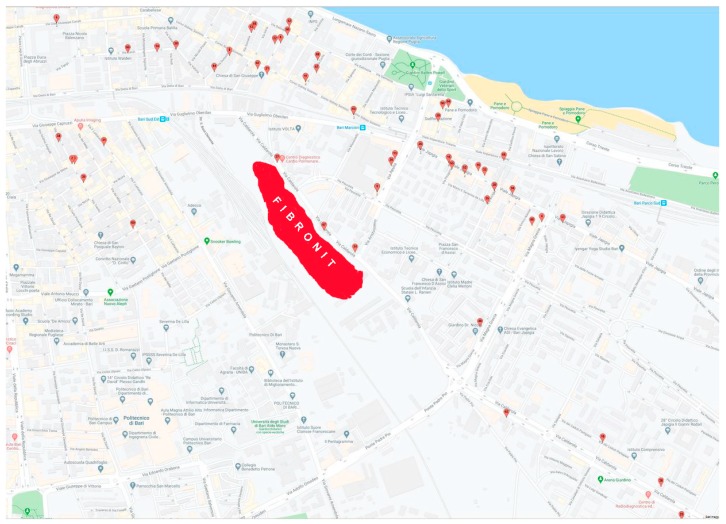
Graphic representation of the Fibronit area and all the addresses of the 71 MM cases.

Of the total number of MM cases among Bari residents, 9.85% had the only environmental exposure linked to the proximity of the residence, within 3000 m, to the Fibronit factory (Figure 1).

All 71 subjects with documented environmental exposure at diagnosis were still living in Bari. Thirty-one males (43.6%) and 40 females (56.3%) represented a M/F ratio of 0.8. The subjects had pleural MM, with 56 subjects having the epithelioid histotype, 7 with the fibrous (sarcomatoid) histotype and 8 with the biphasic histotype, and only three of these subjects had peritoneal mesothelioma, all with the epithelioid histotype (Table 2, Table 3, Table 4, Table 5 and Table 6).

The average age at diagnosis was 65.0 years for males (range 50–89) and 65.2 for females (range 37–90).

The average age at the start of exposure was 15.3 years for males (range 0–44) and 14.6 years for females (range 0–45).

The average latency was 50.7 years for males (range 23–73) and 51.1 years for females (range 20–81).

The average length of residence within 3000 m from the factory was 29.7 years for males (range 7–64) and 31.0 for females (range 4–71).

### 3.2. Comparison between Morphological Types

We compared the individual characteristics of the epithelioid mesothelioma type with those of the other two types (biphasic and fibrous (sarcomatoid)) because we wanted to study the association between the various types. The results of the univariate approach highlights four high odds ratios for females (OR = 1.304(0.442–3.845)) for subjects living at a distance of 550–1000 m (OR = 2.025(0.382–10.736)) or at a distance lower than or equal to 550 m (OR = 1.800 (0.328–9.879)), for subjects with an exposure length longer than 21 years (21–40 years) (OR = 1.767 (0.488–6.395)); >40 years (OR = 4.437 (0.559–35.238)) and for subjects with a latency length of 46–55 years (OR = 1.641(0.388–6.934)) (Supplementary Materials Table S4). All these odds are strong but not statistically significant.

### 3.3. Multivariate Analysis

The adjusted risk (adjusted OR) of observing the epithelial form of mesothelioma among subjects living at short distances from Fibronit was high (OR = 1.870 (0.353–9.905)) for people living at distances of 550–1000 m and for those living at distances less than 550 m (OR = 1.470 (0.262–8.248)). Additionally, the subjects with a high length of exposure showed a relevant risk of epithelioid mesothelioma for both 21–40 years of exposure (OR = 2.027 (0.521–7.890)) and more than 40 years of exposure (OR = 2.879 (0.651–12.736)). Additionally, the trends were high (Table 7). All the estimates were high but not significant because of the low power of this transitional study. The adjustment for latency showed the same trend (Table 8).

### 3.4. Cluster Analysis

**Figure 2:**
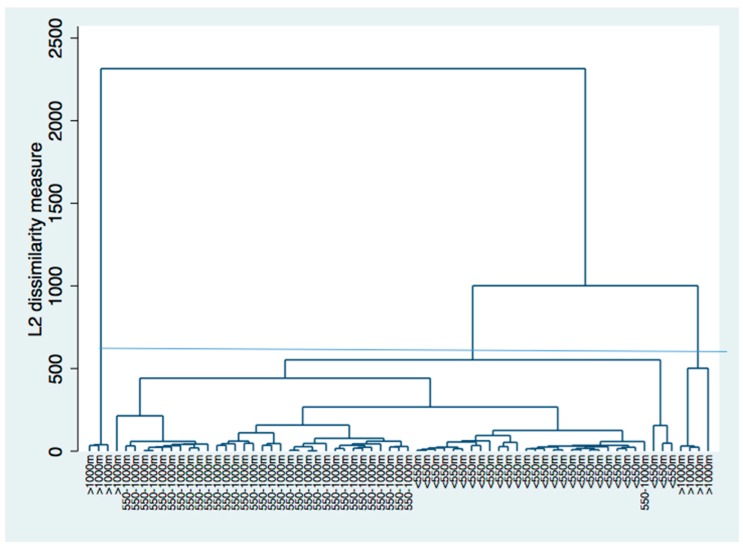
Dendrogram for sex exposure and latency of the cluster analysis. Two main clusters are evident, corresponding to the people resident at a distance of >1000 m and another corresponding to people living at a distance of <1000 m (<500 m and 500–1000 m).

The dendrogram of the cluster analysis of sex, exposure and latency of the cohort of mesothelioma subjects shows the greatest level of dissimilarity (>500 L2) for subjects living at distances greater than 1000 m from the site. The subjects living at distances less than 1000 or 550 m showed the highest similarity of the studied variables (Figure 2) (Supplementary Materials Figure S1).

### 3.5. Principal Component Analysis (PCA)

**Figure 3:**
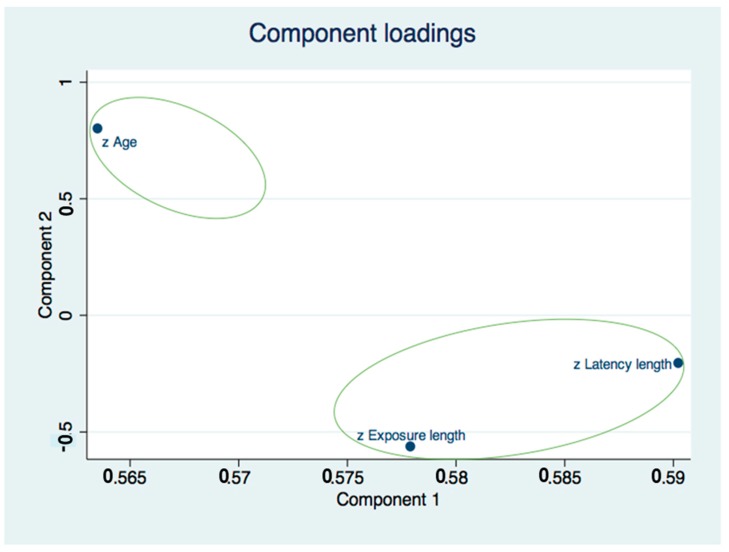
Component loading plot demonstrating two main components. Two main components are present: the first of the age variable and the second of the time related variables (exposure length and latency length).

The component loading plot demonstrated two main components: the first consisted of age, and the second consisted of exposure length and latency length (Figure 3). The likelihood ratio (LR) test for independence (X^2^ = 18.21; *p* ≤ 0.0004) and sphericity (X^2^ = 18.35; *p* ≤ 0.0004) were both statistically significant (Supplementary Materials Tables S2 and S3). The principal component analysis highlights eigenvalues that are statistically significant (eigenvalue = 1.623 (1.089–2.156)) for component 1 (Supplementary Materials Tables S5 and S6).

## 4. Discussion

It is possible to discuss these results starting from the fact that there is agreement in the scientific community that it is not possible to set a threshold value for exposure to asbestos, below which there is no risk of mesothelioma [41]. Moreover, the risk of MM increases with the intensity, duration, and frequency of exposure to asbestos [12,41].

The findings of the Italian MM incidence surveillance system, ReNaM, documented that 4.4% of the 21,387 MM cases (1993–2015) were due to environmental exposure [42]. The ARM data for the years 1993–2018 reported a total of 2236 MM cases with 6.5% resulting from environmental exposure, of which 48.3% were for Bari residents due to the asbestos cement factory (Fibronit). These data are in agreement also with Fazzo et al. [30], who reported significant MM clusters (*p* < 0.10) corresponding to areas that hosted major asbestos cement plants (Casale Monferrato, Broni, Bari). Using detailed information collected by the Apulia regional mesothelioma registry, this study provided evidence of a continuing health impact of the Fibronit asbestos cement factory in Bari on the local community.

Indeed, this study included cases with a definite diagnosis confirmed by immunochemistry, and over 92% of cases included in the study had direct interviews by means of the standardized questionnaire used by the Italian mesothelioma registry (ReNaM) [36].

Nine cases, or 12.6%, were between 37 and 50 at the age of diagnosis. The anticipation of the age of onset can be typical of residential exposure and was associated with cumulative exposure [41,43]. Indeed, these data agree with that of the age at the first exposure, which occurred at birth for 21 or 29.5% of the subjects and between the ages of 2 and 25 years for 32 or 45% of the cases [43]. Dalsgaard et al. recently [21] found a significantly increased risk of MM after environmental asbestos exposure during childhood. The second Italian Consensus Conference on mesothelioma stated that the risk of MM increases with cumulative exposure even at low levels, lung fiber burden and time since exposure gives more weight to exposure that occurred early [44]. Additionally, the M/F ratio is in agreement with the MM cases from environmental exposure as well as latency and age at first exposure [18].

The data on latency are strongly connected to this finding, with 15 or 21.1% of the cases between 20 and 40 years, while the latency is between 41 and 81 years in 56 cases or 78.8% of the cases, as is typical of cases with environmental exposure. These data are consistent with previous evidence of a long latency period and a median latency of 48 years [15,42,43].

Regarding the year of diagnosis, only 26 (36.6%) cases were ascertained from 1989 to 2000, while from 2001 to 2019, the number of environmental cases reached 45 (63.3%). If we consider the first year of exposure, 51 or 71.8% of the cases were first exposed between 1935, the year when the plant was established, and 1959, while the danger of asbestos began to be discussed in 1960 [45].

In agreement with the previous studies [33,34] with reference to the linear distance between the source of exposure, which was the Fibronit plant in this study, 31 or 43.6% of the cases were residents that lived within a radius of 500 m, and 33 or 46.4% of the cases lived between 500 and 1000 m, while only 7 or 9.8% of the cases lived between one thousand and 3000 m from the factory. Residential history and distance from Fibronit have been considered proxies for environmental exposure to asbestos.

Considering the duration of the exposure, i.e., residence near the factory, 25 subjects, or 35.2%, experienced between 4 and 20 years of environmental exposure; in contrast, for 46 subjects, or 64.7%, the exposure was between 21 and 71 years. Compared to this cumulative exposure, this value is a useful summary exposure index, although estimation of cumulative dose is difficult, especially for retrospective evaluation of exposure [12]; moreover, for environmental exposure, it is important to understand that it is not always a low level of exposure [46]. For this cluster, the estimated dose, which is the inhaled fibers that are deposited or retained, could be estimated by default based on the data in Napoli et al. [39] as 24,282.72 fibers/day, although it is a very rough estimate of lifetime exposure. This estimate was indirectly confirmed by the quantitative analysis of fibers in the lung tissue of a patient in this cluster where there were 2,300,000 fibers x gram of dry lung tissue [47]. In a 2012 study [47], a high pulmonary fiber load was described in five cases of mesothelioma, two women and three men; after an accurate reconstruction of the circumstances of exposure, these subjects were determined to not be professionally exposed but instead they were residents near Fibronit. The subjects aged between 36 and 65 at diagnosis, who were diagnosed between 2005 and 2009, lived for periods between 2 and 24 years between 1960 and 1997 at distances between 200 and 2000 m from the factory. The pulmonary load of fibers ranges from 110,000 to 2,300,000 fibers per gram of dry lung (f/g). In two cases, a 51-year-old woman and a 53-year-old man, concentrations greater than 1,000,000 f/g of amphibole fibers were found, which is the value that was proposed as a cut off to identify subjects with occupational exposure to asbestos, even when no evidence of such exposure was present in their work histories. A linear relationship between lung load and environmental exposure indices based on the distance between the residence and the factory has been demonstrated [47].

The exposure index at the bottom level corresponds to an average cumulative exposure of less than 0.1 fibers/mL-y, an average concentration of approximately 0.1 fibers/l, as reported by the monograph of the International Agency for Research on Cancer (IARC) no. 100 [1]. In addition, the WHO has estimated that with continuous exposure at 0.4–1 fibers/l, the risk of MM would be (4 to 10) × 100,000. The linear extrapolation to 0.1 fibers/l (the current background level) would correspond to an excess in the order of one case (from 0.4 to 2.5) of MM per 100,000 people (Regional Office of the World Organization of Health for Europe, 2000) [48].

A recent case-control study [49] explored the relationship between cumulative exposure and pleural MM after nonoccupational exposure, studied the risk associated with asbestos materials in residential areas, and determined a cumulative exposure index to estimate exposure frequency, duration and intensity. The study showed a relationship between MM pleural risk and cumulative exposure after environmental nonoccupational exposure (OR = 2.0 95% CI 1.2–3.2) and confirmed the quantitative relationship between MM incidence and cumulative exposure. Asbestos exposure, even at low levels of exposure, is often due to nonoccupational exposures (OR = 3.8 95% CI 1.3–11.1).

Additionally, the features of the three cases of peritoneal mesothelioma of this cluster confirm that the risk increases according to the power of the time since first exposure (TSFE) [50] and are in agreement with previous studies [51,52].

In addition, in relation to an interaction between genetic risk factors and asbestos in the development of MM [53], two family (consanguineous) groups were present in this cluster: a mother and daughter who both resided for 11 years near Fibronit [47] and a mother and son who resided near the factory for more than 24 years.

Using detailed information collected by the regional mesothelioma registry, this study provided evidence of the continuing health impact of the Fibronit asbestos cement factory in Bari on the resident population, as also described in other similar contexts [54,55].

Studying the environmental risk of MM is challenging due to the long latency period and the small number of cases, and this is one limit of this study together with the lack of availability of analytical data on the concentration of asbestos fibers in urban air from 1935–2018. Furthermore, this type of exposure is involuntary and is unknown in most cases [14]. In this context, due to the often unknown environmental exposure to asbestos, low price biomarkers of exposure are needed [56,57,58,59] for the ascertainment of environmental exposure and the early prevention of health effects. From this point of view, a new frontier can be represented by the use of the comet assay [60,61,62,63] since inhaled asbestos may induce oxidative stress and biochemical changes in lipids, proteins, DNA and RNA [18].

Epidemiological surveillance of incident cases of mesothelioma at national and international levels is recognized to be of primary importance for understanding the health damage resulting from exposure to asbestos, the identification of exposure circumstances still present in the area and the evolution of “own” or “improper” exposures to asbestos, such as for the activation of mechanisms to protect patients and their families. The registration of mesothelioma cases is an essential tool for the development of epidemiological knowledge and support for research activities. The registration is a tool for risk control and prevention and an indicator to guide the choices and organization of health services in terms of public health and population needs [64,65,66]. From the experience of the city of Bari, it is also clear that since these are environmental exposures and considering the interest of citizens for the protection of public health, the development of specific communication interventions is essential during the risk management process. Psychological support interventions in communities affected by mesothelioma are particularly important [67,68]. The asbestos emergency in the city of Bari also highlights the importance of Environmental Health Literacy (EHL) on environmental health or community health, which can be defined as the ability to search, understand, evaluate and use information on public health, and the importance of the environment to encourage the adoption of informed choices, reduce health risks, and improve the quality of life and the environment. For this reason, it is necessary to adopt a communication strategy that involves different stakeholders, health workers, authorities, local communities, and the media that presents the results of environmental epidemiology research that are useful for health interventions and the promotion of healthy activities that actively involve the whole community in a participatory process [69,70]. Public health can directly pursue the public good in terms of the maximum benefit for the greatest number of subjects, or it may have a privileged consideration for the worst situations [71].The public health relevance of environmental exposure to asbestos in contaminated sites of national interest, such as Bari, was also highlighted in the final report from the Government Conference on Asbestos and Asbestos-Related Diseases (ARDs) [31,72]. The development of national programs to eliminate ARDs was a point of the final declaration of the Sixth Ministerial Conference on Environment and Health of the WHO European Region, which aimed to achieve the health and well-being objectives of the United Nations 2030 Agenda for Sustainable Development [73].

## 5. Conclusions

The past use of asbestos has generated serious consequences for public health among the inhabitants of Bari, and the results shown here confirm the association between MM and environmental pollution in the city of Bari. It should also be emphasized that the scientific support of the series of cases collected by our regional register and the continuous documentation of the effects of environmental exposure to asbestos have increased the awareness among the citizens of Bari and have prompted the authorities to plan the remediation of sites to safeguard public health. In light of current scientific knowledge, it is essential to adopt precautionary principles to pursue the best solutions with respect to local priorities and the specific needs associated with the reduction in the health impact of involuntary asbestos exposures through timely remediation and surveillance agreed upon by the international scientific community.

Future extension of our study with an analytical ad hoc study to compare MM case data with different types of asbestos exposure and calculate the relative incidence rates will be worthwhile.

## Figures and Tables

**Table 1:** Main characteristics of the mesothelioma cohort.

Variables	Categories	Frequency	Percent	Cumulative
Sex	Females	40	56.34	
	Men	31	43.66	100.00
Age	≤65 years	37	52.11	
	>65 years	34	47.88	100.00
Morphological Types	Biphasic	8	11.26	
	Epithelioid	56	78.87	
	Fibrous (sarcomatoid)	7	9.85	100.00
Distances from Fibronit	>1000 m	7	9.85	
	550–1000 m	33	46.47	
	≤500 m	31	43.66	100.00
Exposure length	≤20 years	25	35.21	
	21–40 years	22	30.98	
	>40 years	24	33.80	100.00
Latency length	≤45 years	24	33.80	
	46–55 years	22	30.98	
	>55 years	25	35.21	100.00
Total		71	100.00	

**Table 2:** Peritoneal MM cases.

Peritoneal MM	Case 1	Case 2	Case 3
Sex	male	male	female
Year of diagnosis	1999	2001	1999
Age at diagnosis	62	73	67
Year of first exposure	1936	1970	1935
Age at first exposure	from birth	42	3
Years of exposure	62	31	64
Latency	62	31	64

**Table 3:** The overall distribution by age at diagnosis and sex is as follows.

Age Years	Male	Female	Total
37–50	1	8	9
51–60	8	9	17
61–70	11	8	19
71–80	7	9	16
81–90	4	6	10
total	31	40	71

**Table 4:** The overall distribution of age at the start of exposure and sex is as follows.

Age at First Exposure	Male	Female	Total
birth	8	13	21
2–15	7	11	18
16–25	5	9	14
26–40	9	6	15
41–45	2	1	3
total	31	40	71

**Table 5:** The overall distribution by latency and sex is as follows.

Latency in Years	Male	Female	Total
20–29	2	1	3
31–40	3	9	12
41–50	9	9	18
51–60	10	11	21
61–70	6	5	11
71–81	1	5	6
total	31	40	71

**Table 6:** The overall distribution by duration of exposure and sex is as follows.

Duration of Exposure Years	Male	Female	Total
4–10	4	6	10
11–20	8	7	15
21–30	10	6	16
31–40	2	4	6
41–50	5	9	14
51–60	1	4	5
61–71	1	4	5
total	31	40	71

**Table 7:** Adjusted risks (ORs) of epithelioid mesothelioma by distance from Fibronit and exposure length.

Variables	Dummy	Odds Ratio	LCI (95%) *	UCI (95%) *
**Distance from Fibronit**	>1000 m	1.000	-	-
	550–1000 m	1.870	0.353	9.905
	≤550 m	1.470	0.262	8.248
**Sex**	Males	1.000	-	-
	Females	1.129	0.360	3.536
**Age Class**	≤65 years	1.000	-	-
	>65 years	0.847	0.254	2.824
**Exposure Length**	<21 years	1.000	-	-
	21–40 years	2.027	0.521	7.890
	>40 years	2.879	0.651	12.736

* LCI: low confidence interval; UCI: upper confidence interval.

**Table 8:** Adjusted risks (ORs) of epithelioid mesothelioma by distance from Fibronit and latency length.

Variables	Dummy	Odds Ratio	LCI (95%) *	UCI (95%) *
**Distance from Fibronit**	>1000 m	1.000	-	-
	550–1000 m	2.038	0.371	11.190
	≤550 m	1.771	0.320	9.805
**Sex**	Males	1.000	-	-
	Females	1.337	0.445	4.015
**Age Class**	≤65 years	1.000	-	-
	>65 years	1.085	0.333	3.535
**Latency Length**	≤45 years	1.000	-	-
	46–55 years	1.634	0.376	7.097
	>55 years	1.207	0.312	4.666

* LCI: low confidence interval; UCI: upper confidence interval.

## Supplementary Materials

The following are available online at https://www.mdpi.com/1660-4601/17/8/2636/s1, Figure S1: Scree plot showing high Eigenvalues (>1) only for the first two components. Table S1: The overall distribution by year of incidence and sex, Table S2: The overall distribution of the calendar years of the start of exposure and sex, Table S3: The overall distribution by residence distance from the factory and sex, Table S4: Univariate Risk (Ors) Epithelioid mesotheliomas by different variables, Table S5: Principal components/covariance of the studied variables, Table S6: Distributions of Eigenvalues by components.
