# Supplementary material for: Asbestos Air Pollution: Description of a Mesothelioma Cluster Due to Residential Exposure from an Asbestos Cement Factory
Source: Int J Environ Res Public Health. 2020 Apr 12;17(8):2636. doi: 10.3390/ijerph17082636 (PMC7215593; doi:10.3390/ijerph17082636)
Supplement: Supplementary file 1 [file ijerph-17-02636-s001.zip › Supplementary Materials/CR7YR9X8_D358-EA82-4308-24F3-1E7A.pdf]

SPRINGER NATURE
Author Services

Editing Certificate

This document certifies that the manuscript

Asbestos air pollution: description of a mesothelioma cluster due to residential exposure from an asbestos cement factory.

prepared by the authors

Vimercati L., Cavone D., Delfino C., Caputi A., De Maria L., Sponselli S., Corrado V., Ferri G. and Serio G.

was edited for proper English language, grammar, punctuation, spelling, and overall style by one or more of the highly qualified native English speaking editors at SNAS.

This certificate was issued on **March 16, 2020** and may be verified on the SNAS website using the verification code **D358-EA82-4308-24F3-1E7A**.

Neither the research content nor the authors' intentions were altered in any way during the editing process. Documents receiving this certification should be English-ready for publication; however, the author has the ability to accept or reject our suggestions and changes. To verify the final SNAS edited version, please visit our verification page at secure.authorservices.springernature.com/certificate/verify.
If you have any questions or concerns about this edited document, please contact SNAS at support@as.springernature.com.

SNAS provides a range of editing, translation, and manuscript services for researchers and publishers around the world.
For more information about our company, services, and partner discounts, please visit authorservices.springernature.com.
