# Supplementary material for: Asbestos Air Pollution: Description of a Mesothelioma Cluster Due to Residential Exposure from an Asbestos Cement Factory
Source: Int J Environ Res Public Health. 2020 Apr 12;17(8):2636. doi: 10.3390/ijerph17082636 (PMC7215593; doi:10.3390/ijerph17082636)
Supplement: Supplementary file 1 [file ijerph-17-02636-s001.zip › Supplementary Materials/Supplementary Material Figure 1.doc]

**Supplementary material Figure 1.** Scree plot showing high Eigenvalues (>1) only for the first two components.
