# Supplementary material for: Asbestos Air Pollution: Description of a Mesothelioma Cluster Due to Residential Exposure from an Asbestos Cement Factory
Source: Int J Environ Res Public Health. 2020 Apr 12;17(8):2636. doi: 10.3390/ijerph17082636 (PMC7215593; doi:10.3390/ijerph17082636)
Supplement: Supplementary file 1 [file ijerph-17-02636-s001.zip › Supplementary Materials/Supplementary Material Table 1.doc]

**Supplementary materials Table 1.** The overall distribution by year of incidence and sex is as follows:

| Calendar years | male | female | total |
| --- | --- | --- | --- |
| 1989-90 | 1 | 1 | 2 |
| 1991-95 | 4 | 5 | 9 |
| 1996-2000 | 8 | 7 | 15 |
| 2001-5 | 4 | 11 | 15 |
| 2006-10 | 6 | 4 | 10 |
| 2011-15 | 5 | 7 | 12 |
| 2016-19 | 3 | 5 | 8 |
| total | 31 | 40 | 71 |
