# Supplementary material for: Asbestos Air Pollution: Description of a Mesothelioma Cluster Due to Residential Exposure from an Asbestos Cement Factory
Source: Int J Environ Res Public Health. 2020 Apr 12;17(8):2636. doi: 10.3390/ijerph17082636 (PMC7215593; doi:10.3390/ijerph17082636)
Supplement: Supplementary file 1 [file ijerph-17-02636-s001.zip › Supplementary Materials/Supplementary Material Table 2.doc]

**Supplementary materials Table 2.** The overall distribution of the calendar years of the start of exposure and sex is as follows:

| Calendar years | male | female | total |
| --- | --- | --- | --- |
| 1935-39 | 3 | 6 | 9 |
| 1940-49 | 7 | 6 | 13 |
| 1950-59 | 12 | 17 | 29 |
| 1960-69 | 5 | 8 | 13 |
| 1970-79 | 4 | 3 | 7 |
| total | 31 | 40 | 71 |
