# Supplementary material for: Asbestos Air Pollution: Description of a Mesothelioma Cluster Due to Residential Exposure from an Asbestos Cement Factory
Source: Int J Environ Res Public Health. 2020 Apr 12;17(8):2636. doi: 10.3390/ijerph17082636 (PMC7215593; doi:10.3390/ijerph17082636)
Supplement: Supplementary file 1 [file ijerph-17-02636-s001.zip › Supplementary Materials/Supplementary Material Table 3.doc]

**Supplementary materials Table 3.** The overall distribution by residence distance from the factory and sex is as follows:

| Distance in meters | male | female | total |
| --- | --- | --- | --- |
| 0-500 | 13 | 18 | 31 |
| 501-1000 | 15 | 18 | 33 |
| 1001-1500 | 1 | 3 | 4 |
| 1501-3000 | 2 | 1 | 3 |
| total | 31 | 40 | 71 |
