# Supplementary material for: Asbestos Air Pollution: Description of a Mesothelioma Cluster Due to Residential Exposure from an Asbestos Cement Factory
Source: Int J Environ Res Public Health. 2020 Apr 12;17(8):2636. doi: 10.3390/ijerph17082636 (PMC7215593; doi:10.3390/ijerph17082636)
Supplement: Supplementary file 1 [file ijerph-17-02636-s001.zip › Supplementary Materials/Supplementary Material Table 4.doc]

| **Supplementary material Table 4.** Univariate Risk (Ors) Epithelioid mesotheliomas by different variables. | | | | | | | | | | | | |  |
| --- | --- | --- | --- | --- | --- | --- | --- | --- | --- | --- | --- | --- | --- |
|  |  | Epithelioid Mesothelioma | | |  |  |  |  |  |  |  |  | |
|  |  | Yes | No | Total |  |  |  |  |  |  |  | | |
| Variables | Categories | n (%) | n (%) | n (%) | **OR** | LCI | UCI | chi2 | p | Trend chi2 | P | | |
| Sex | Males | 22 (41.51) | 9 (50.00) | 31 (43.66) | **1.000** |  |  |  |  |  |  | | |
|  | Females | 31 (58.49) | 9 (50.00) | 40 (56.34) | **1.304** | 0.442 | 3.845 | 0.23 | 0.629 | 0.390 | 0.533 | | |
| Age class | <=65 years | 10 (55.56) | 28 (52.83) | 38 (53.52) | **1.000** |  |  |  |  |  |  | | |
|  | > 65 years | 8 (44.44) | 25 (47.17) | 33 (46.48) | **1.116** | 0.378 | 3.296 | 0.040 | 0.842 | 0.040 | 0.840 | | |
| Distance from FIBRONIT | > 1000 m | 5 (9.43) | 3 (16.67) | 8 (11.27) | **1.000** |  |  |  |  |  |  | | |
|  | 550-1000 m | 27 (50.94) | 8 (44.44) | 35 (49.3) | **2.025** | 0.382 | 10.736 | 0.720 | 0.397 |  |  | | |
|  | <= 500 m | 21 (39.62) | 7 (38.89) | 28 (39.44) | **1.800** | 0.328 | 9.879 | 0.470 | 0.493 | 0.200 | 0.650 | | |
| Exposure length | <=20 years | 16 (30.19) | 9 (50.00) | 25 (35.21) | **1.000** |  | . |  |  |  |  | | |
|  | 21-40 years | 17 (32.08) | 5 (27.78) | 22 (30.99) | **1.767** | 0.488 | 6.395 | 0.770 | 0.379 |  |  | | |
|  | > 40 years | 20 (37.74) | 4 (22.22) | 24 (33.80) | **4.437** | 0.559 | 35.238 | 2.380 | 0.123 | 2.310 | 0.128 | | |
| Latency length | <=45 years | 16 (30.19) | 7 (38.89) | 23 (32.39) | **1.000** |  |  |  |  |  |  | | |
|  | 46-55 years | 15 (28.30) | 4 (22.22) | 19 (26.76) | **1.641** | 0.388 | 6.934 | 0.460 | 0.497 |  |  | | |
|  | > 55 years | 22 (41.51) | 7 (38.89) | 29 (40.85) | **1.375** | 0.396 | 4.775 | 0.250 | 0.615 | 0.230 | 0.628 | | |
|  | Total | 53 (100.00) | 18 (100.00) | 71 (100.00) |  |  |  |  |  |  |  | | |
