# Supplementary material for: Asbestos Air Pollution: Description of a Mesothelioma Cluster Due to Residential Exposure from an Asbestos Cement Factory
Source: Int J Environ Res Public Health. 2020 Apr 12;17(8):2636. doi: 10.3390/ijerph17082636 (PMC7215593; doi:10.3390/ijerph17082636)
Supplement: Supplementary file 1 [file ijerph-17-02636-s001.zip › Supplementary Materials/Supplementary Material Table 5.doc]

| **Supplementary material Table 5.** Principal components/covariance of the studied variables. | | | | | | | | | |  |
| --- | --- | --- | --- | --- | --- | --- | --- | --- | --- | --- |
|  | Principal components/covariance | | | |  | N of obs | 71 |  | | |
|  |  |  |  |  |  | N of comp | 2 | | | |
|  |  |  |  |  |  | Trace | 3 | | | |
|  |  |  |  |  |  | Rho | 0.779 | | | |
|  | SEs assume multivariate normality | | | |  | SE(Rho) | 0.036 | |  | |
|  |  | Coef. | Std. Err. | z | P>z | [95% Conf. | Interval] | | | |
| **Eigenvalues** | Comp1 | 1.623 | 0.272 | 5.960 | 0.000 | 1.089 | 2.156 | | | |
|  | Comp2 | 0.714 | 0.120 | 5.960 | 0.000 | 0.479 | 0.948 | | | |
| **Comp1** | Z Age | 0.564 | 0.115 | 4.880 | 0.000 | 0.337 | 0.790 | | | |
|  | Z Exposure class | 0.578 | 0.110 | 5.270 | 0.000 | 0.363 | 0.793 | | | |
|  | Z Latency | 0.590 | 0.104 | 5.660 | 0.000 | 0.386 | 0.795 | | | |
| **Comp2** | Z Age | 0.798 | 0.360 | 2.220 | 0.027 | 0.093 | 1.503 | | | |
|  | Z Exposure class | -0.566 | 0.967 | -0.590 | 0.558 | -2.461 | 1.329 | | | |
|  | Z Latency | -0.208 | 1.281 | -0.160 | 0.871 | -2.719 | 2.304 | | | |
| LR test for independence (X^2^ 18.21; p<= 0.0004) | | | | | | | | | |  |
| LR test for sphericity (X2 18.21; p<= 0.0004) | | | | | | | | | |  |
