# Supplementary material for: Asbestos Air Pollution: Description of a Mesothelioma Cluster Due to Residential Exposure from an Asbestos Cement Factory
Source: Int J Environ Res Public Health. 2020 Apr 12;17(8):2636. doi: 10.3390/ijerph17082636 (PMC7215593; doi:10.3390/ijerph17082636)
Supplement: Supplementary file 1 [file ijerph-17-02636-s001.zip › Supplementary Materials/Supplementary Material Table 6.doc]

| **Supplementary material Table 6.** Distributions of Eigenvalues by components. | | | | | | |  |
| --- | --- | --- | --- | --- | --- | --- | --- |
|  | Eigenvalues | Proportions | SE Proportions | Cumulative | SE Cum | Bias | |
| Comp1 | 1.623 | 0.541 | 0.051 | 0.541 | 0.051 | 0.034 | |
| Comp2 | 0.714 | 0.238 | 0.038 | 0.779 | 0.036 | 0.116 | |
| Comp3 | 0.664 | 0.221 | 0.036 | 1.000 | 0.000 | -0.150 | |
